# Correction: Ortiz-Islas et al. Evolution of Alzheimer’s Disease Therapeutics: From Conventional Drugs to Medicinal Plants, Immunotherapy, Microbiotherapy and Nanotherapy. *Pharmaceutics* 2025, *17*, 128

**DOI:** 10.3390/pharmaceutics17121625

**Published:** 2025-12-18

**Authors:** Emma Ortiz-Islas, Pedro Montes, Citlali Ekaterina Rodríguez-Pérez, Elizabeth Ruiz-Sánchez, Talía Sánchez-Barbosa, Diego Pichardo-Rojas, Cecilia Zavala-Tecuapetla, Karla Carvajal-Aguilera, Victoria Campos-Peña

**Affiliations:** 1 Laboratorio de Neurofarmacologia Molecular y Nanotecnologia, Instituto Nacional de Neurología y Neurocirugía, Manuel Velasco Suárez, Mexico City 14269, Mexico; emma.ortiz@innn.edu.mx (E.O.-I.); crodriguez@innn.edu.mx (C.E.R.-P.); 2 Laboratorio de Neuroinmunoendocrinología, Instituto Nacional de Neurología y Neurocirugía, Manuel Velasco Suárez, Mexico City 14269, Mexico; pedrovolvox@gmail.com; 3 Laboratorio de Neuroquímica, Instituto Nacional de Neurología y Neurocirugía, Manuel Velasco Suárez, Mexico City 14269, Mexico; ruizruse@yahoo.com.mx; 4 Laboratorio Experimental de Enfermedades Neurodegenerativas, Instituto Nacional de Neurología y Neurocirugía, Manuel Velasco Suárez, Mexico City 14269, Mexico; talisabar@hotmail.com (T.S.-B.); cztecua@yahoo.com.mx (C.Z.-T.); 5 Departamento de Biomedicina Molecular, Centro de Investigación y de Estudios Avanzados del Instituto Politécnico Nacional, Mexico City 07360, Mexico; 6 Programa Prioritario de Epilepsia, Instituto Nacional de Neurología y Neurocirugía, Manuel Velasco Suárez, Mexico City 14269, Mexico; diego.pichardo@uabc.edu.mx; 7 Laboratorio de Nutrición Experimental, Instituto Nacional de Pediatría, Mexico City 04530, Mexico; karla_ca@yahoo.com

**References**

The original reference 253 has been retracted and its contents are no longer of reference value. The authors would like to make the following corrections for their published paper [1]:

Deleting original reference 253:

253. Gao, C.; Chu, X.; Gong, W.; Zheng, J.; Xie, X.; Wang, Y.; Yang, M.; Li, Z.; Gao, C.; Yang, Y. Neuron tau-targeting biomimetic nanoparticles for curcumin delivery to delay progression of Alzheimer’s disease. *J. Nanobiotechnol.* **2020**, *18*, 71. https://doi.org/10.1186/s12951-020-00626-1.

With this correction, the order of some references has been adjusted accordingly. The authors state that the scientific conclusions are unaffected. This correction was approved by the Academic Editor. The original publication has also been updated.
